# Editorial: Transplantation and cellular therapy in lymphomas and plasma cell disorders

**DOI:** 10.3389/fonc.2024.1527836

**Published:** 2024-12-10

**Authors:** Saad Z. Usmani, Nilanjan Ghosh, Peter Voorhees, Edward Copelan

**Affiliations:** ^1^ Myeloma Service, Department of Medicine, Memorial Sloan-Kettering Cancer Center, New York, NY, United States; ^2^ Atrium Health Levine Cancer Institute, Wake Forest University School of Medicine, Charlotte, NC, United States

**Keywords:** hematopoietic cell transplantation, CAR T cells, lymphomas, multiple myeloma, cellular therapy

We, the editors of this Research Topic of Frontiers in Oncology, invite readers to examine the 13 expert perspectives provided on these timely topics. Hematopoietic cell transplantation, particularly autologous transplantation, has long been a standard of care in the treatment of many individuals with lymphomas or multiple myeloma. The safety and effectiveness of the procedure has improved in recent years and in this issue authorities in these malignancies detail its evolving, but critical, role in the context of the growing application of chimeric antigen receptor (CAR)-based therapies.

While allogenic transplantation was the first successful cellular therapy to treat hematologic malignancies, it is a rather blunt instrument and associated with significant toxicities and only modest effectiveness in lymphomas and multiple myeloma. Allotransplant’s effectiveness relies on donor cells recognizing and attacking antigens on tumor cells which do not invoke autoreactivity. Most antigens expressed on malignant cells are also expressed on normal cells. The immune system has developed to avoid autoimmune reactions. Incorporation of synthetic receptors, derived from immunoglobulin to redirect T cell specificity, and the zeta chain from CD3, to activate function, produced first generation CAR T-cells (1), which evaded this tolerance (2).

The addition of costimulatory domains provided for expansion of functional CARs and persistence of these tumor-fighting cells (3). Clinical trials with CD19-directed CAR Ts showed durable complete responses in substantial proportions of patients with B cell malignancies including non-Hodgkin lymphoma (4) and subsequent trials confirmed these exceptional responses. Subsequently, dramatic responses to BCMA CAR T cells in multiple myeloma were demonstrated (5). In both lymphomas and multiple myeloma, the role of CAR Ts has assumed a growing role and moved steadily towards an earlier role in management.

This Research Topic details the appropriate role of CAR Ts and also details their toxicities, including B cell aplasia, cytokine release syndrome, neurotoxicity, and infections. Perhaps most importantly, we include discussions of the obstacles to global implementation of CAR T therapy with a focus on disparities in access and of challenges to development of off-the-shelf CAR Ts. We hope this Research Topic provides better understanding of the appropriate use of transplantation and CART Ts at present and insight into the future.
